# Sanitizing agents for virus inactivation and disinfection

**DOI:** 10.1002/viw2.16

**Published:** 2020-05-24

**Authors:** Qianyu Lin, Jason Y. C. Lim, Kun Xue, Pek Yin Michelle Yew, Cally Owh, Pei Lin Chee, Xian Jun Loh

**Affiliations:** ^1^ Soft Materials Department Institution of Materials Research and Engineering Agency for Science Technology and Research (A*STAR) Innovis Singapore; ^2^ NUS Graduate School for Integrative Sciences and Engineering National University of Singapore Singapore

**Keywords:** disinfectant, sanitizer, surface, virucidal, virus

## Abstract

Viral epidemics develop from the emergence of new variants of infectious viruses. The lack of effective antiviral treatments for the new viral infections coupled with rapid community spread of the infection often result in major human and financial loss. Viral transmissions can occur via close human‐to‐human contact or via contacting a contaminated surface. Thus, careful disinfection or sanitization is essential to curtail viral spread. A myriad of disinfectants/sanitizing agents/biocidal agents are available that can inactivate viruses, but their effectiveness is dependent upon many factors such as concentration of agent, reaction time, temperature, and organic load. In this work, we review common commercially available disinfectants agents available on the market and evaluate their effectiveness under various application conditions. In addition, this work also seeks to debunk common myths about viral inactivation and highlight new exciting advances in the development of potential sanitizing agents.

## Graphical abstract

Virus pandemics are recurrent in human history, and one of the major means of human‐to‐human viral transmissions occur through contact with contaminated surfaces. Herein, the authors provide a summary of the major commercially available sanitizing agents used to inactivate viruses and critically evaluate their effectiveness to do so.

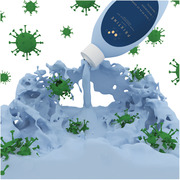

## 1 INTRODUCTION

Viral transmissions and infections have posed severe threats to human health and well‐being throughout history, and have led to widespread socioeconomic disruptions. During the 2014 Ebola pandemic in West Africa, the gross domestic product (GDP) growth of Liberia, one of the worst‐affected countries, fell from 8.7% in 2013 to just 0.7% in 2014.^1^ Bolstered by the close‐knit global connectivity we enjoy today, the threat of a global virus pandemic is greater now than any other time in human history, as viruses can spread across the globe at unprecedented rates. Even a century ago in 1918, the “Spanish influenza” pandemic caused a worldwide healthcare catastrophe, with more than 50 million deaths and 500 million infections.^2^ A similar pandemic today will undoubtedly result in even more disastrous outcomes.^3^ At the time of writing in early April 2020, the novel coronavirus causing Covid‐19 (SARS‐CoV‐2, or formerly known as HCoV‐19), which was first reported by China in late 2019, has resulted in more than a million confirmed cases of infections and almost 57,000 fatalities worldwide.^4^ This is the largest coronavirus outbreak in human populations within the first 20 years of the 21st century, occurring on a larger scale than the earlier outbreaks of Severe Acute Respiratory Syndrome (SARS) and Middle East Respiratory Syndrome (MERS) in 2002–2004 and 2012, respectively.^5^ While government interventions can influence the rates and range of outbreaks,^6^ individuals can play equally, or arguably even more important roles in limiting the spread of viruses in the public and healthcare arenas.^7^ Human‐to‐human transmission of common influenza viruses and coronaviruses can occur through virus‐laden body fluids, as well as self‐innoculation of the mucous membranes in the nose, mouth, or eyes by touching contaminated dry surfaces.^8^ Depending on the type of surface and ambient conditions, viruses can persist for as short as <5 min to greater than 28 days on inanimate surfaces.^9^ The use of sanitizing agents for personal care and surface disinfection are clearly of paramount importance in limiting viral transmissions, by inactivating the viruses before they have a chance to enter the human body.

In this review, we summarize the various types of sanitizing agents used in commercially available formulations scientifically demonstrated for their virucidal properties to inactivate viruses in suspension and on surfaces. Viruses that require vectors for transmission, such as the chikungunya and dengue viruses, are not considered. These “virucidal” agents can either destroy viruses or alter their surface structures to prevent them from infecting potential host cells (Section 2). They differ from “antiviral” compounds^10^ which inhibits virus replication in host cells. The effective dose of each sanitizing agent, exposure time for effective virucidal activity, suitability for usage under domestic or healthcare/hospital settings and mechanisms of action are considered. We also explore what has been scientifically shown for some common myths believed to inactivate viruses and prevent their spread. Finally, we present promising new research directions, materials, and strategies that have been shown to inactivate viruses but have yet to reach widespread commercial availability.

## 2 GENERAL WORKING PRINCIPLES OF DISINFECTANTS AGAINST VIRUSES

### 2.1 Viruses and infectivity

Viruses are typically composed of a viral capsid containing nucleic acids inside. The nucleic acid serve as the template information for replication, while the capsid and its associated proteins function both to protect the nucleic acid and bind to host cell receptors.^11^ The coronavirus SARS‐CoV‐2, for example, uses the spike glycoprotein to bind to host cell sialic acid receptors.^12^ Outside of a host cell, viruses are unable to replicate and increase in number. However, they can often survive for long periods of time in this state.^13^ When they encounter a suitable host cell, they will infect and enter the host cell, hijacking the cellular machinery for its own replication. Viruses are able to infect cells, including even bacterial cells, and cause a range of commonly seen diseases (see Table 1). This is exacerbated by the lack of effective treatment against several of these viruses.

**TABLE 1:** Viruses and common diseases

Name of virus	Category of disease	Disease
Influenza	Respiratory	Flu
Coronavirus	Respiratory	Cold (mostly)
Herpes Simplex virus 2	Sexually transmitted infections	Herpes
Human Immunodeficiency virus (HIV)	Immune	Acquired immune deficiency syndrome (AIDS)
Norovirus	Gastrointestinal	Vomiting/diarrhea
Hepatitis A virus	Gastrointestinal	Liver inflammation
Poliovirus	Neurological	Polio
Adenovirus	Respiratory, ocular, gastrointestinal	Cold, viral conjunctivitis vomiting, and diarrhea

### 2.2 Surfaces spread viruses

Surfaces, including our hands, play an important part in the spread of viruses. Viruses such as poliovirus and bacteriophage showed a much higher survival when they were transferred by direct contact of surfaces, as opposed to droplet aerosolization or dust containing viruses.^14^ Hand‐to‐surface contact of only 5 s was sufficient to transfer a significant proportion of the virus, and viruses could then spread by touching the mucosa of the nose or conjunctiva of the eye.^15^ The chance of spread is in turn directly correlated with the viral survival time on the surface, which shows considerable variation between different viruses. For example, among different soft surfaces tested, enteric viruses were shown to survive on wool blankets for the longest period of time, poster card for the shortest period of time, and cotton fabric for an intermediate period of time.^13^ A very recent study has found that the Covid‐19 coronavirus (SARS‐CoV‐2) can persist longest on propylene plastic surfaces and stainless steel, with viable viruses found up to 72 hr after initial application though at a greatly reduced viral titer.^16^ Much shorter persistence was observed on copper surfaces, with no viable viruses observed after 4 hr. While the closely‐related SARS‐CoV‐1 coronavirus showed no significant statistical differences in half‐life on these surfaces, SARS‐CoV‐2 could persist considerably longer on cardboard surfaces: 24 hr were required for no viable SARS‐CoV‐2 to be detected, compared with just 8 hr for SARS‐CoV‐1.

### 2.3 Factors affecting disinfectant efficacy

The main measure of efficacy of the disinfectant is by the fold reduction in infectivity of the virus, and this is typically conducted by carrier tests and suspension tests. The key parameters that affect the efficacy of disinfection agents against viruses include the contact time, concentration of disinfection agent, and the particular virus involved.^17^ The disinfection inactivation can be described by modified forms of the Chick–Watson law:

$$\log\frac{N}{N_{o}}=-kCT$$

where *N*_o_ is the original number of microbes, *N* is the final number of microbes, *C* is the disinfectant concentration, *T* is the contact time, and k is the inactivation rate constant (specific to the microbe).^18^ With a decrease in the concentration of the active disinfecting ingredient, the contact time will likely need to be increased to achieve adequate disinfection.^19^ A reduction of viral infectivity by 4 log units correlates to a 99.99% reduction in the viral titer. The log unit reduction and percent (%) reduction are used interchangeably in literature to describe disinfectant efficacy.

Disinfection efficacy can also be influenced by environmental factors. If disinfection requires chemical reactions to take place, such as for formaldehyde, then the rate of disinfection will be higher at higher temperatures. Under cold temperatures, certain disinfectants could be ineffective as the rate of disinfection would be exceedingly low.^20^ Humidity is also another factor that could affect disinfectant penetration to the virus. For reactions such as aldehyde disinfectants, a change in pH will also affect the disinfectant efficacy.^17^

### 2.4 Factors influencing virus susceptibility

There are specific unique characteristics of viruses that influence inactivation by disinfection. There are three main types of viruses with different structures, classified here according to increasing difficulty of chemical disinfectant inactivation: enveloped viruses, large unenveloped viruses, and small unenveloped viruses (see Table 2). Larger viruses are generally more sensitive to disinfectants, although there are exceptions.^21^ A few disinfectant solutions tested were all effective against the enveloped viruses Herpes Simplex Virus and Human Immunodeficiency Virus (HIV) type 1, although less effective against the small non‐enveloped human coxsackie virus.^22^ Enveloped viruses contain a lipid envelope that is required for infection, and therefore interfering with the envelope could potentially reduce virus infectivity. Lipophilic disinfectants can often be used to inactivate enveloped viruses. In contrast, non‐enveloped viruses utilize a protein coat for infection, and therefore inactivation often requires denaturation of the redundant viral capsid proteins or essential replicative proteins.^23^ Disinfectants that disrupt proteins such as glutaraldehyde or sodium hypochlorite could be effective at inactivating non‐enveloped viruses.^21^ Sodium hypochlorite was shown to inactivate the bacteriophage PAO1, and further electron microscopy studies showed that there was extensive structural damage to the phage, including damage to capsid proteins.^24^ However, the disinfectant might also need to penetrate to destroy the nucleic acids, as viruses such as polio retain infectivity with the RNA alone.^21^ While the enveloped virus Influenza H1N1 could be inactivated by all the disinfectants tested,^25^ it is much more challenging to inactivate small non‐enveloped noroviruses, and several commonly available disinfectants are not able to sufficiently reduce infectivity.^26^, ^27^

**TABLE 2:** Types of common viruses and overall resistance to disinfectants. Table modified from reference^21^

Type of virus^[a]^	Common examples	Resistance to disinfectants
Enveloped	Herpes Simplex Virus, Human immunodeficiency virus (HIV), Influenza, Coronavirus	Low
Large non‐enveloped	Adenovirus	Medium
Small non‐enveloped	Poliovirus, coxsackievirus, parvovirus, norovirus	High

Viruses also show resistance to disinfection due to the cellular materials that viruses are normally associated with. Viruses are normally reliant on host cells for replication, so they are often found together with material such as cell debris, soil, and aerosolized droplets.^20^ These are called viral clumping protective factors, and they can both reduce the penetration of the disinfectant to the virus, and can also interact and reduce the activity of the disinfecting agents. This has a big impact on the disinfectants, necessitating a much higher concentration for effective disinfection. Disinfection is commonly associated with and reliant on cleaning processes, as the removal of organic material impurities first can allow for a better disinfection process.^28^ Viruses can also aggregate in the environment, for example, upon exposure to disinfectants, thereby making it more difficult for disinfectants to penetrate and access the viruses.^29^

## 3 COMMERCIALLY AVAILABLE VIRUCIDAL SANITIZING AGENTS

### 3.1 Alcohols

Alcohols, specifically isopropyl alcohol (otherwise known as isopropanol and propan‐2‐ol) and ethyl alcohol (ethanol), are capable of inactivating a wide spectrum of bacterial, fungi, and viral activities. These actives play an important role in the healthcare industry for skin antisepsis and disinfecting small medical tools. Although it is shown to be effective in annihilating infectious microorganisms, alcohols are not sporicidal^30^, ^31^ and are often coupled with other major biocidal actives to improve its disinfection efficacy.^31^

Potent biocidal agents eradicate viruses and bacteria by various mechanisms such as disrupting the structure of the cell, and coagulating and/or denaturing proteins in the microorganisms. Although few studies have been done to fully understand the biocidal activity of alcohol, it is often believed that alcohols disrupts the cell membrane and denatures the proteins in general.^31^, ^32^ Viruses as well as many other microorganisms are commonly susceptible to this mode of action. Previous studies have reported that with the inclusion of water in the biocidal system, the efficacy of alcohol increases as water would facilitate quicker denaturing of proteins.^30^, ^31^, ^33^ Additionally, the inclusion of water significantly increase the effectiveness of the alcohols as it delays the evaporation of the alcohol and increase its exposure to viruses and bacteria.

Generally, the efficacy of alcohols in eradicating microorganisms is optimum between 60% and 90% by solution in water (v/v).^30^, ^33^ In one of the studies, it was reported that 80% ethyl alcohol was effective at eradicating hepatitis B virus under 2 min and 70% isopropyl alcohol of that within 10 min.^34^ However, the virucidal activity of alcohol depends greatly on its concentration of the actives and the type of test viruses. Ethyl alcohol is effective against enveloped viruses and a few non‐enveloped viruses. Studies have shown that ethyl alcohol inactivates enveloped virus such as herpes and influenza to select non‐enveloped viruses such as adenovirus, rhinovirus, and rotavirus.^30^ Isopropyl alcohol, however, was reported to be effective against enveloped viruses but ineffective against similar non‐enveloped viruses.^30^ The differential virucidal action could potentially be a result of the lipophilic nature of isopropyl alcohol as compared to ethanol.^31^ The efficacy of alcohols to inactivate viruses is heavily dependent on the surface properties of the microorganism. Isopropyl alcohol, by nature lipophilic, interacts favorably with enveloped viruses and disrupts their activity effectively. The envelope layer of viruses mainly comprises lipid bilayers, which causes the membrane to be sensitive to the chemical and physical conditions.^35^ Non‐enveloped viruses are generally known to be more resilient to disinfectants compared to enveloped viruses, and this includes alcohols. Studies have been shown that both alcohols are potent virucidal agents against enveloped viruses such as Hepatitis B virus^34^, Herpes virus^36^,SARS‐CoV,^37^ and human immunodeficiency virus (HIV)^38^, but not non‐enveloped viruses such as Hepatitis A virus^34^ and polio virus.^39^

Although alcohols are effective in eradicating some types of viruses, other disinfectants such as quaternary ammonium compounds (QAC), glutaraldehyde, and hydrogen peroxide quickly outshine its performance.^30^ Therefore, disinfectants with alcohol as its main active ingredients are generally not used to disinfect critical equipment or environment in the healthcare settings.^30^ The use of isopropyl alcohol is also limited as it only inactivates lipid viruses. This greatly reduces the capabilities of alcohol as a broader use disinfecting agent. Because alcohols are flammable liquids, large amounts of alcohol will increase the risks and dangers of it as a disinfecting agent. The flash point of a higher concentration of alcohol solution is lower than that of a lower concentration.^32^, ^40^ According to Boyce, 70% ethyl alcohol has a flash point at 20.5°C, while the flash point of 30% ethyl alcohol is at 29°C.^32^ Furthermore, prolonged and repeated usage of alcohol compromises the integrity of materials such as plastics and dyes. Materials with constant exposure to alcohol may experience discoloration, cracking, and swelling due to the effects of alcohol. Another difficulty with the use of alcohol is that it evaporates quickly when exposed to air, therefore reducing the contact time with the virus. Achieving maximum disinfection is difficult unless the tools are immersed in a bath for a period of time.

Even though the capabilities of alcohol are limited, the active is still commonly used in various disinfectant procedures. It is imperative to note that together with other properties of alcohol, its role as a disinfectant is still irreplaceable. Alcohols are often used as an effective disinfectant for thermometer, non‐critical tools, and non‐invasive probes in the hospital^30^. Non‐critical surfaces of medical instruments that are reusable are also disinfected with alcohol. Another advantage of using alcohol as a disinfectant is that it is user friendly. Alcohol solutions are non‐staining, evaporates quickly, low toxicity compared to other forms of disinfectant, and have a mild acceptable odor. These characteristics are critical in the healthcare settings as it contributes to the efficiency and the necessary sanitization in the system.

### 3.2 Surfactants

Surfactants are amphiphilic moieties possessing both hydrophilic and lipophilic segments^41^ and are further classified into cationic,^42^ anionic,^43^ non‐ionic,^44^ and zwitterionic^45^ surfactants. They are often the active ingredients found in household disinfectants and detergents and have been demonstrated to be capable of inactivating viruses.^46^, ^47^, ^48^ Due to their amphiphilic nature, their main mechanism of viral disinfection is usually solvating and disrupting the lipid‐based envelope of the virus.^46^ Enveloped viruses such as the family of coronaviruses, among which they include SARS‐CoV‐1,^49^ MERS,^50^ and the new SARS‐CoV‐2 viruses,^51^ are thus susceptible to these surfactants. However, some of the surfactants do not rely on solvating lipid envelopes to inactivate viruses. Surfactants such as sodium lauryl sulfate (SLS) possess strongly hydrophilic heads that readily target the capsid proteins, unfolding, and extracting them before gradually solubilizing lipid membranes.^43^

#### 3.2.1 Cationic surfactants (Quaternary Ammonium Compounds)

**FIGURE 1:**
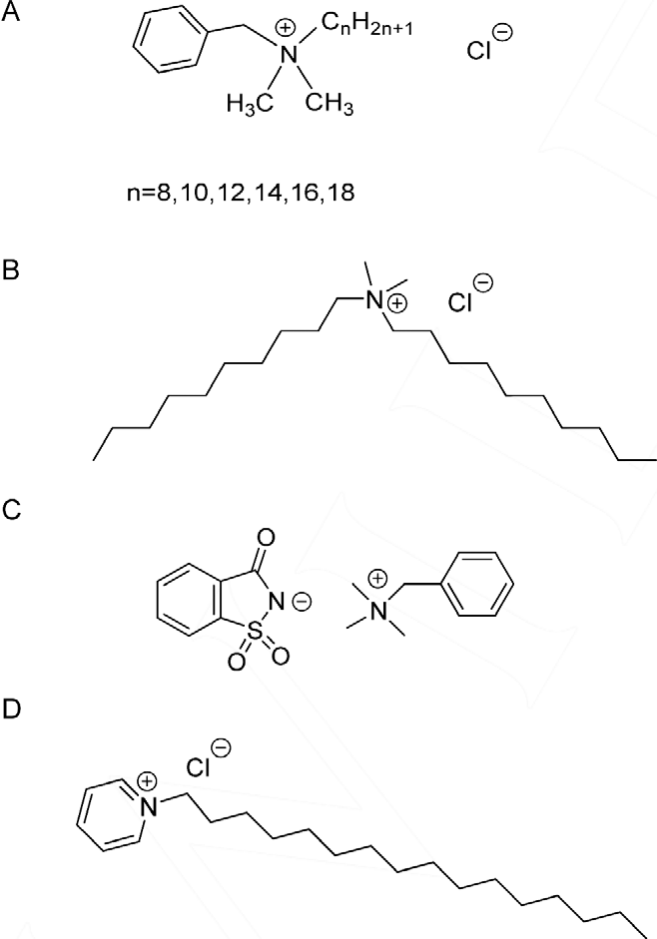
Chemical structures of (A) benzalkonium chloride, (B) didecyldimethyl ammonium chloride, (C) alkyl dimethyl benzyl ammonium saccharinate, and (D) cetyl pyridinium chloride

Quaternary ammonium compounds (QACs) form the main bulk of the cationic surfactants^52^ and they mostly inactivate viruses by solvating and disrupting lipid envelopes or membranes^46^, ^53^. They are characterized by the presence of a cationic ammonium group which is the hydrophilic head.^53^ The ammonium group has four organic substituents such as alkyl or heterocyclic groups forming the lipophilic tail and the charge is balanced by an anion such as a halide or sulfate.^54^ Some of the most common QACs found in household disinfectants include benzalkonium chloride,^42^ didecyldimethyl ammonium chloride,^46^ alkyl dimethyl benzyl ammonium saccharinate,^55^ and cetyl pyridinium chloride^56^ (Figure 1). In addition to the common QACs, a new generation of QACs has also been developed and they are referred to as twin‐chain or dialkyl quaternaries, examples include didecyl dimethyl ammonium bromide^57^ and dioctyl dimethyl ammonium bromide.^58^ They claim to retain virucidal activity better in hard water and also in the presence of anionic residues.^59^

QACs are attractive as they are relatively nontoxic, colorless, and odorless.^60^ They are well‐known for inactivating enveloped viruses but their virucidal activity depends on concentration, duration of application, and temperature.^42^, ^61^ Tsujimura et al. evaluated the virucidal effect of three QACs, namely benzalkonium chloride, (BZK), mono; bis (tri‐methyl ammonium methylene chloride)‐alkyl (C_9–15_) toluene (MBAT), and didecyldimethyl ammonium chloride (DDA).^46^ It is found that the QACs require warmer temperatures to exhibit more significant virucidal properties.^46^, ^62^ The QACs at their highest recommended concentrations of 0.05% (w/v), 0.02% (w/v), and 0.02% (w/v), respectively, had no virucidal effect on enveloped equine herpesvirus type 1 after 10 min reaction time at 0°C.^46^ When the temperature is increased to room temperature, the virucidal activity of the QACs is found to be dependent upon duration of reaction.^46^ Reaction times shorter than 1 min produced no virucidal effect, while the minimum effective concentration (MEC) at 5 min is mostly double that of the MEC for a 10 min reaction.^46^ MEC is defined as the lowest concentration of the biocide that reduced the virus titer value by 99.99% or greater as compared to control reactions.^46^ This suggests that effective disinfection utilizing QACs is best achieved using warm water and longer reaction times.^62^

An advantage of utilizing QAC‐based disinfectants is their relatively high tolerance toward the presence of contaminating organic matter; their ability to inactivate viruses is usually not diminished by the presence of organic matter^46^, ^63^ as seen in other common disinfectants such as alcohol and chlorine based disinfectants.^46^ Tsujimura et al. demonstrated that the virucidal effects of MBAT and DDA are unchanged by the addition of 5% fetal bovine serum (FBS) but the MEC of BZK increased by four times when exposed to 5% FBS.^46^ However, in another study by Rabenau et al.,^64^ the authors demonstrated that the virucidal effect of BZK based disinfectants against SARS coronavirus is not significantly reduced by the presence of albumin or sheep erythrocytes. The seemingly contrasting results might be attributed to the fact that BZK disinfectant formulations tested by Rabenau et al. contain other active ingredients like glutaraldehyde and didecyldimonium chloride too.^64^

QACs are regarded as lipophilic and target enveloped viruses by solvating their lipid‐based envelope.^17^ However, non‐enveloped viruses are mainly characterized as possessing a protein capsid which is hydrophilic,^17^ thus QACs may have lower affinity and also lower disinfectant ability toward these viruses.^17^, ^43^, ^65^ It is possible that the cationic moiety interacts with the protein capsid on the non‐enveloped viruses and lead to viral inactivation.^22^, ^43^, ^65^ Romanowski et al. investigated the virucidal effectiveness of benzalkonium chloride (BZK) against common human ocular types of adenovirus that are non‐enveloped viruses.^42^ The results suggest that the effectiveness of BZK varies with the virus and also concentration of BZK. At the highest tested concentration of 0.1% BZK, five adenoviruses are inactivated with virus titers >3Log_10_ units while the remaining two adenoviruses are insufficiently inactivated with reduced titers >1Log_10_ units but <3Log_10_ units. Lower concentrations of BZK exhibited compromised ability to inactivate the adenoviruses. In another study by Wood and Payne, a formulation with a final BZK concentration of 0.2% (w/v) is found to be effective against non‐enveloped human coxsackie virus after 1 min of exposure but was ineffective toward inactivating other more resistant non‐enveloped viruses like poliovirus and human adenovirus under the same conditions.^22^ The same BZK formulation is also found to inactivate only some of the enveloped virus like the herpes simplex virus but failed to inactivate other enveloped viruses like human immunodeficiency virus and human coronavirus after 1 min of exposure.^22^ This shows that the virucidal effects of QACs are highly dependent upon the specific virus and the conditions of exposure to achieve virus inactivation needs to be tailored.

#### 3.2.2 Anionic surfactants

**FIGURE 2:**
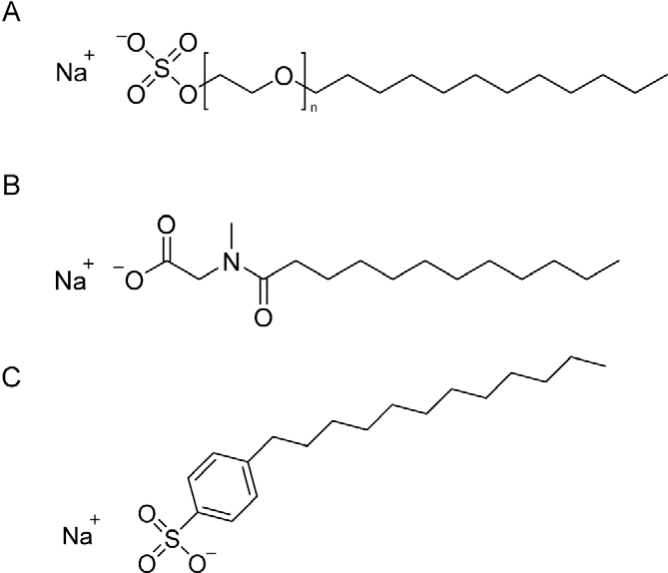
Chemical structures of (A) sodium laureth sulfate, (B) *N*‐lauroylsarcosine and (C) sodium linear alkylbenzene sulfonate

Sodium laureth sulfate, *n*‐lauroylsarcosine, and sodium linear alkylbenzene sulfonate (Figure 2) are some common anionic surfactants present in detergent and personal‐care products such as soaps, shampoos, and toothpaste,^66^ and they have been demonstrated to be effective disinfectants towards a range of viruses.^48^, ^67^ In the study by Tsujimura et al., the authors demonstrated that the disinfection ability of sodium linear alkylbenzene sulfonate (LAS) against enveloped equine herpesvirus type 1 is dependent upon duration, temperature, and organic matter contamination.^46^ LAS is able to effectively inactivate equine herpesvirus type 1 at 0°C when applied at 0.05% concentration for 10 min. The minimum effective concentration (MEC) is reduced by four times when the temperature is raised to room temperature for 10 min. However, when the duration is shortened to 1 min, the MEC rose to >0.05%. In addition, MEC value at room temperature and 10 min reaction duration increased by two times in the presence of 5% contaminating FBS.

Although the main mode of disinfection by surfactants is usually via solvating the lipid envelopes of viruses,^17^ surfactants like sodium laureth sulfate (SLS) that possess strongly hydrophilic heads have a different disinfection mechanism as compared to a more hydrophobic surfactant like *N*‐lauroylsarcosine (LS).^43^ The interaction between SLS and viruses is dominated by ionic interactions instead of hydrophobic interactions.^43^ SLS thus solubilizes liposomal membranes at a slow rate but bind rapidly to the protein component of Ca^2+^‐ATPase membranes causing unfolding and extraction of the proteins.^43^ The viral penetration into cells can be divided into three stages, the first being the initial attachment of the virus to the cell surface heparin sulfate, followed by forming stable attachment to the heparin sulfate, and then fusion of the virus with the cell membrane leading to penetration of the virus into the cell.^43^ Piret et al. report that SLS and LS reduce viral infection of cells by interfering with different steps of the viral penetration process.^43^ Incubating enveloped herpes simplex virus with LS reduced the first stage initial binding to cells while SLS functioned by disrupting the fusion process between the viral envelope and cell membrane. As LS reduces the initial binding of the virus to cells, the virucidal effectiveness of LS requires the pre‐incubation of the virus with LS in order to prevent future viral binding to cells. The lack of a pre‐incubation period significantly reduced the virucidal effectiveness of LS. On the other hand, as SLS does not reduce initial viral binding to cells, the virucidal effects of SLS is less reliant upon pre‐incubation of the virus with SLS. Moreover, it is also shown that SLS can exhibit viral inactivation effects on viruses already firmly attached to cells. Due to the complementary mechanisms of disinfection, the authors suggested synergistic combination of SLS and LS in a single formulation. In addition, the authors also note that the presence of contaminating organic matter reduced the disinfectant ability of both SLS and LS and consequently concentrations 5 and 2.5 times higher respectively are required to achieve viral inactivation.

#### 3.2.3 Non‐ionic and zwitterionic surfactants

**FIGURE 3:**
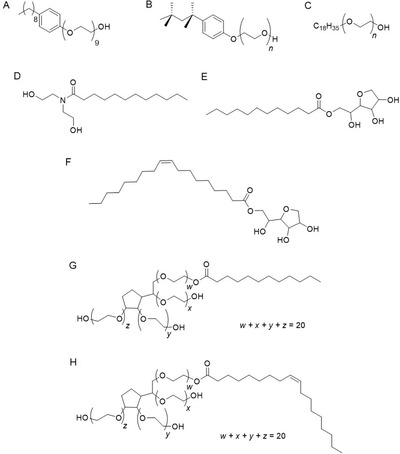
Chemical structures of non‐ionic surfactants. (A) Nonoxynol‐9, (B) Triton X‐100, (C) Brij‐97 contain ether linkages. (D) Onyxol 345 contains amide linkage. (E) Span‐20 and (F) Span‐80 contain ester linkages. (G) Tween‐20 and (H) Tween‐80 contain ether‐ester linkages

Non‐ionic surfactants are commonly used as emulsifiers and can be classified by the type of bonds between the hydrophobic and hydrophilic segments.^44^, ^68^, ^69^ The connecting bonds are commonly amide, ether, ether–ester, or ester bonds.^44^, ^69^, ^70^ Nonoxynol‐9 (nonylphenoxypolyethoxyethanol), Triton X‐100 (*p*‐diisobutylphenoxy polyethoxyethanol), and Brij‐97 (polyoxyethylene (10) oleyl ether) are non‐ionic surfactants containing ether bonds (Figure 3A–C) while Onyxol 345 (N,N‐bis(2‐hydroxyethyl)dodecanamide) contains amide linkages^44^, ^70^ (Figure 3D). Examples of non‐ionic surfactants containing ester bonds are such as Span‐20 (sorbitan monolaurate) and Span‐80 (sorbitan monooleate)^44^ (Figure 3E,F). Lastly, Tween‐20 (polysorbate‐20) and Tween‐80 (polysorbate‐80) are non‐ionic surfactants containing ether–ester linkages^44^ (Figure 3G,H). These non‐ionic surfactants inactivate viruses by solvating the viral envelope and disrupting the nucleocapsid.^44^ Their viral disinfectant ability is highly associated with the type of linkages between their hydrophobic and hydrophilic segments.^44^ Non‐ionic surfactants with ether and amide linkages have high virucidal effectiveness while those containing ester and ether–ester linkages are much weaker at inactivating viruses when tested under the same conditions.^44^ However, the high virucidal activity of the ether and amide containing non‐ionic surfactants also corresponded to high cytotoxicity and would thus require high dilution factors.^44^

**FIGURE 4:**
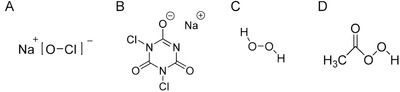
Chemical structures of (A) Sodium hypochlorite (B) Sodium dichloroisocyanurate (C) Hydrogen peroxide (D) Peracetic acid

Zwitterionic surfactants are molecules bearing both cationic and anionic charges but with an overall neutral charge.^71^ Their virucidal activity is less investigated but they may possess interesting properties. Crawford et al. demonstrated that a zwitterionic detergent, Empigen BB®, an alkylbetaine based on a C12‐C14 alcohol, is able to inactivate influenza A and B but still retain the biological activity of the surface haemagglutinin (HA) and neuraminidase (NA) antigens.^45^ The authors proposed that the mechanism of disinfection by the zwitterionic detergent is via viral disruption instead of solubilizing the surface proteins as cationic, anionic, and non‐ionic surfactants do.^45^, ^72^ This special ability to inactivate viruses but retain the biological activity of their surface antigens allows the zwitterionic detergent to be utilized during the development of vaccines.^45^

### 3.3 Oxidizing agents

Disinfectants such as sodium hypochlorite, hydrogen peroxide, and peracetic acid utilize their oxidizing capability to inactivate viruses. For the small non‐enveloped viruses such as noroviruses that are difficult to disinfect, strong oxidizing agents are among the most effective disinfectants.^73^

#### 3.3.1 Sodium hypochlorite

Sodium hypochlorite, the active ingredient in household bleach, is a strong oxidizing agent. It dissolves in water to form hypochlorous acid, which can be reduced to form water and the chloride anion. The hypochlorous acid molecule can react with peptide bonds and thiol groups, chemically oxidizing proteins and other biomolecules and abolish function.^74^ The efficacy of disinfection decreases with an increase in pH, likely due to the decreased proportion of hypochlorous acid group present.^75^

Sodium hypochlorite is fast acting, and is effective at low concentrations. At 10 ppm available chlorine, sodium hypochlorite is able to inactivate bacteriophage PAO1 within 30 s, and further characterization studies showed that a large number of structural components were damaged or deformed by the treatment. Generally, it was found that the extent of damage was proportional to the concentration of sodium hypochlorite and the contact time.^24^ Sodium hypochlorite at 0.5% (5000 ppm) could inactivate Ebola surrogate bacteriophage Phi6 with 10 min contact time on a stainless steel surface, but not a nitrile surface.^28^ Sodium hypochlorite was able to inactivate adenovirus together with 5% serum at a concentration of 1900 ppm with 1 min contact time, achieving a mean log_10_ reduction of infectivity of 4.87.^76^

Sodium hypochlorite can be used for difficult to disinfect non‐enveloped viruses such as noroviruses. Sodium hypochlorite could inactivate Norwalk virus, a type of norovirus, at a concentration of 160 ppm after 30 s exposure, as quantified by RT‐PCR.^77^ Sodium hypochlorite could also inactivate the norovirus surrogates, feline calicivirus and murine norovirus, to greater than a 5 log10 reduction of infectivity with a concentration of 2700 ppm and 1 min.^78^ Sodium hypochlorite at 0.5% concentration with 1 min contact time was able to deactivate the Hepatitis A virus, while another oxidizing agent peracetic acid was unable to do so.^79^

Sodium hypochlorite at high concentrations of 1000 ppm is regularly used for clinical disinfection. However, it has an odor and can be irritating to mucous membranes at high concentrations. Less concentrated hypochlorous acid solutions are less stable to environmental factors such as temperature and light, and the oxidizing potential can be used up by impurities.^80^ In terms of application, a concentrated stock of 5% sodium hypochlorite that is more stable is typically recommended to be diluted 100× to 0.05% before use so as to mostly avoid the downsides of concentrated sodium hypochlorite during the disinfection process. The most common oxidizing agent disinfectant in the United States is aqueous solutions of 5.25–6.15% sodium hypochlorite, also known as household bleach.^81^

Sodium hypochlorite is sensitive to the presence of organic material, and a significantly higher concentration is required to achieve the same disinfectant efficacy. While 100 ppm sodium hypochlorite can effectively disinfect a clean surface of HIV‐1 virus in 30 s, the activity is reduced in the presence of organic material. A higher concentration of 500 ppm and 1–2 min is required in the presence of 80% serum, and around 10,000 ppm (1%) is required in the presence of 80% blood.^82^ Sodium hypochlorite at 50 ppm was found to disinfect HIV in saline alone, but a 50‐fold higher concentration of 2,500 ppm was required to disinfect in the presence of 10% plasma, and 5,000 ppm (1%) was required in the presence of blood.^83^ In a review guidance for clinicians, noncritical surfaces that are contaminated with blood or other tissues are suggested to be cleaned first to remove organic material before spot decontaminating with sodium hypochlorite solution.^84^

#### 3.3.2 Sodium dichloroisocyanurate

Compared to sodium hypochlorite, sodium dichloroisocyanurate has disinfectant activity that persists for longer, is more tolerant to the presence of organic material, and has a higher disinfectant efficacy overall. Sodium dichloroisocyanurate could inactivate the bacteriophage Phi6, an Ebola surrogate, at 0.5% concentration in 10 min on multiple different surfaces, while sodium hypochlorite could not inactivate sufficiently on nitrile surface.^28^ Sodium dichloroisocyanurate at 50 ppm was found to disinfect HIV in saline alone, but a 50‐fold higher concentration of 2500 ppm was required to disinfect in the presence of 10% plasma, and 5,000 ppm (0.5%) was required in the presence of blood.^83^ Sodium dichloroisocyanurate at 10,000 ppm shows disinfectant activity in the presence of 70% serum, while sodium hypochlorite only shows similar activity in presence of 20% serum.^85^ This is postulated to be because only 50% of the total chlorine is in the available hypochlorite/hypochlorous acid component form that can be neutralized by serum, while the rest of the chlorine can be progressively pushed toward hypochlorite/hypochlorous acid to maintain the chemical equilibrium.

#### 3.3.3 Hydrogen peroxide

Hydrogen peroxide is a strong broad spectrum inactivation agent. It decomposes to form water, oxygen and the highly reactive hydroxyl free radicals, which can cleave or crosslink a large range of biomolecules including proteins, nucleic acids and lipids.^17^ A 13% solution with a 5 min contact time was able to inactivate to greater than 5 log_10_ reduction both the enveloped virus Herpes Simplex virus, and the non‐enveloped virus poliovirus, showing a similar broad spectrum virucidal activity.^86^. An accelerated hydrogen peroxide‐based (AHP) disinfectant at a concentration of 0.5% and a contact time of 1 min showed a log_10_ reduction in infectivity of over 4 for both enveloped and non‐enveloped viruses, including HIV, coronavirus 229E, poliovirus, and rotavirus (Wa).^87^ The AHP solution at concentration 0.5% was tested with another set of enveloped and non‐enveloped viruses, and showed effective inactivation of the enveloped Sindbis virus and non‐enveloped reovirus within 5 min.^88^

Hydrogen peroxide was also effective against noroviruses, although at generally higher concentrations than sodium hypochlorite. At a concentration of 0.18% and 5 min contact time, the AHP disinfectant was able to inactivate feline calicivirus, and at 3.5% and 10 min contact time, the AHP disinfectant was able to inactivate surrogate murine norovirus to greater than 5log_10_ reduction.^78^ A 2.1% liquid hydrogen peroxide solution with 10 min exposure could inactivate murine norovirus and bacteriophage Phi X174 on stainless steel surfaces by 4 log_10_ units.^89^ A disinfectant comprising of hydrogen peroxide and peracetic acid could inactivate surrogate feline Calicivirus by 99.9% at an effective peroxide concentration of 0.1% and 15 min contact time.^90^

Even when reused, a 7 % solution with a contact time of 5 min was able to show an almost 5 log_10_ reduction in infectivity of poliovirus, showing that some disinfectant activity was still present.^91^ A sonicated hydrogen peroxide system with a cartridge containing hydrogen peroxide at 31.5% and 2 min contact time showed that it could attain a mean log_10_ reduction of 5.20 for human papillomavirus (HPV).^92^

### 3.4 Peracetic acid

Peracetic acid decomposes in an analogous manner to form the highly reactive hydroxyl free radicals, as well as acetic acid and oxygen.^93^ Peracetic acid at 0.2% could inactivate adenovirus 8 in hard water in 5 min, with a mean log_10_ reduction of infectivity of 4.75.^76^ Peracetic acid at 0.23% and at a contact time of 15 min could reduce the infectivity of coliphage MS2 by greater than 3 log_10_ units. There was much more effective deactivation as compared to hydrogen peroxide tested under the same setup.^94^ Peracetic acid at a low concentration of 85 ppm (0.0085%) and contact time of 1 min could reduce the infectivity of a murine norovirus surrogate by 3 log_10_ units in suspension, and reduced virus infectivity on surfaces of fruits and vegetables by 4 log_10_ units.^73^ A peracetic acid and hydrogen peroxide‐based disinfectant, at an effective peroxide concentration of 0.1% and contact time of 15 min could reduce infectivity of feline calicivirus, a norovirus surrogate, by 4 log_10_ units in the absence of feces, while a higher concentration of 2% was required in the “field‐like conditions” with feces as organic material impurity.^90^ Powder forms of peracetic acid at 0.5% concentration and 15 min contact time could reduce the infectivity of porcine epidemic diarrhea virus by more than 99.99% (4 log_10_ units).^95^ These forms of peracetic acid were developed to provide higher stability and which could be dissolved in situ to form the disinfectant solution.

### 3.5 Halogenated compounds

#### 3.5.1 Povidone iodine

**FIGURE 5:**
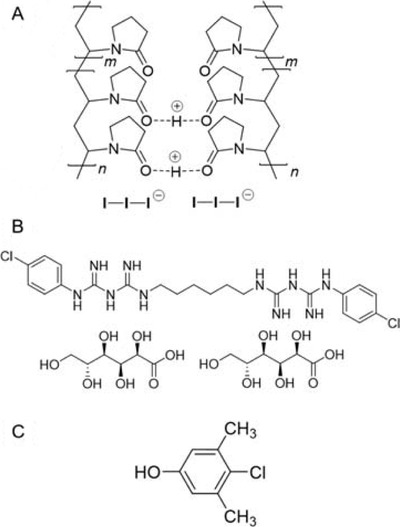
Chemical structures of (A) povidone‐iodine;^110^ (B) chlorhexidine digluconate and (C) chloroxylenol

Povidone iodine is an example of an iodophor, which is a mixture of elemental iodine and a carrier polymer‐ polyvinylpyrrolidone in this case. The carrier polymer has no virucidal activity on its own.^96^ Iodine, the active and powerfully‐virucidal agent, exists as a complex mixture of many species (e.g. I_2_, I^−^, I_3_^−^, IO^−^, IO_3_^−^) at equilibrium in water, but aqueous iodine solutions can be cytotoxic and cause irritancy.^97^ Hence, the polyvinylpyrrolidone polymer entraps the various iodine‐containing species by hydrogen bonding (Figure 5A), providing a reservoir of iodine for its sustained release in small doses at a time, maintaining its virucidal properties for prolonged periods while decreasing toxicity. Povidone‐iodine is a broad‐spectrum virucidal agent manufactured in formulations containing 7.5‐10% iodophor in solution, and are used in clinical applications such as sterilizing agents for pre‐ and post‐operation skin cleaning, in surgical swabs, scrubs and ointments, as well as everyday products including antiseptic handwashes, mouthwashes and gargles that contain lower concentrations of the iodophor.^98^ Povidone‐iodine is not suitable for use with silicone products such as silicone catheters as iodine can cause accelerated deterioration of the material.^99^ The iodophor is capable of inactivating (≥ 4.0 reduction of log_10_ viral infectivity) a large range of viruses which include the enveloped coronaviruses, influenza A^100^ and vaccinia virus, as well as non‐enveloped polyomavirus and adenovirus^101^ within 1 minute (Table 3). For the most‐resistant non‐enveloped polioviruses however, aqueous formulations of povidone‐iodine (Betaisodona ® solution, containing 11% available iodine), required at least an hour for reduction of infectivity of ≥ 4.0log_10_ units. In contrast, alcohol‐containing povidone‐iodine formulations (e.g., Betaseptic Mundipharma®, containing 10% available iodine and ca. 40% 2‐propanol and 40% ethanol) could inactivate these polioviruses within 5 min,^101^ owing to the synergistic virucidal activity of the iodophor and the alcohols. Although it is generally safe and more effective in inactivating viruses than many other antiseptic agents (e.g., benzalkonium chloride, chlorhexidine digluconate),^102^ povidone‐iodine may cause thyroid dysfunction with long‐term prolonged use,^103^ as well as allergic contact dermatitis,^104^ necessitating careful medical monitoring.

**TABLE 3:** Comparison/summary table for virus review in suspension tests without organic load

Class	Sanitizing agent	Safety	Application settings	Advantages/Disadvantages	Concentration (%)	Virus	Exposure time	Reduction of activity (log_10_)^*^ OR Reduction of virus titer (%) OR PFU/ml^×^	Temperature (^o^C)	Ref
Alcohols	Ethyl alcohol	Safe, non‐irritant	Hospital and home settings, personal hygiene	Pros: broad‐spectrum and non‐staining Cons: flammable, requires specific concentration range to be effective	70.0	Poliovirus (Sabin 1an)	1 min	0.4	37	^39^ ^]^
					70.0	Murine Norovirus (CW3)	1 min	>3.6	‐	^137^
					70.0	Murine Norovirus (CW3)	5 min	>3.6	‐	^137^
					70.0	Feline Calicivirus (F9)	1 min	0.5±0.6	‐	^137^
					70.0	Feline Calicivirus (F9)	5 min	2.6±0.3	‐	^137^
					70.0	Human immunodeficient virus (HIV) ‐ I	1 min	>5.50	R.T.	^138^
					70.0	Influenza A (H1N1)	1 min	≥4.84	20	^139^
					60.0	Vaccinia virus strain Lister Elstree (ATCC VR‐1549)	1 min	≥4.38 ± 0.37	20 ‐ 22	^140^
					60.0	Modified vaccinia Ankara strain	1 min	≥5.40 ± 0.36	20 ‐ 22	^140^
					70.0	Mouse hepatitis virus (MHV)	‐	3.92	‐	^40^
					70.0	Transmissible gastroenteritis virus (TGEV)	‐	3.19	‐	^40^
	Isopropyl alcohol	Safe, non‐irritant	Hospital and home settings, personal hygiene	Pros: broad‐spectrum and non‐staining Cons: only inactivates lipid viruses, flammable, requires specific concentration range to be effective,	70.0	Murine Norovirus (CW3)	1 min	2.6±0.3	R.T.	^137^
					70.0	Murine Norovirus (CW3)	5 min	>2.6	R.T.	^137^
					70.0	Feline Calicivirus (F9)	1 min	0.1±0.1	R.T.	^137^
					70.0	Feline Calicivirus (F9)	5 min	0.2±0.2	R.T.	^137^
					60.0	Vaccinia virus strain Lister Elstree (ATCC VR‐1549)	1 min	≥4.38 ± 0.37	20 ‐ 22	^140^
					60.0	Modified vaccinia Ankara strain	1 min	≥5.40 ± 0.36	20 ‐ 22	^140^
Cationic Surfactants – Quaternary Ammonium Compounds	Benzalkonium chloride	Generally safe	Hospital, industrial, household disinfectants	Odorless, colorless, and non‐caustic. Requires warmer temperatures and longer reaction time. Virucidal activity reduced by the presence of contaminating organic matter.	0.2	Human Adenovirus	1 min	0.25	R.T.	^22^
					0.2	Herpes Simplex virus	1 min	>4.51	R.T.	^22^
					0.2	Human Immunodeficiency Virus Type 1	1 min	>1.87	R.T.	^22^
					0.2	Poliovirus	1 min	0.12	R.T.	^22^
					0.2	Human Coxsackie virus	1 min	>5.12	R.T.	^22^
					0.2	Human Coronavirus	1 min	0.0	R.T.	^22^
					0.2	Human Coronavirus ATCC VR‐759 (Strain OC43)	10 min	0.0	R.T.	^22^
					0.05	Murine Hepatitis Virus (Strains MHV‐2 and MHV‐N)	10 min	>3.7	23	^9^
					0.05	Canine coronavirus (Strain 1–71)	10 min	>3.7	23	^9^
					>0.05	Equine herpesvirus type 1	10 min	99%	23 ‐ 25	^44a^
					0.1	Human Adenovirus Type 3	1 h	5.02 ± 1.57	33	^42^
					0.1	Human Adenovirus Type 4	1 h	2.94± 0.80	33	^42^
					0.1	Human Adenovirus Type 5 (Isolate MC)	1 h	5.27 ± 0.73	33	^42^
					0.1	Human Adenovirus Type 5 (Isolate MA)	1 h	3.65 ± 1.04	33	^42^
					0.1	Human Adenovirus Type 7a	1 h	3.71 ± 1.87	33	^42^
					0.1	Human Adenovirus Type 8 (Isolate ED)	1 h	1.80 ± 1.12	33	^42^
					0.1	Human Adenovirus Type 8 (Isolate CR)	1h	1.01 ± 0.50	33	^42^
					0.1	Human Adenovirus Type 19/64	1 h	3.66 ± 1.46	33	^42^
					0.1	Human Adenovirus Type 37	1 h	4.23 ± 0.21	33	^42^
					0.025	Equine herpesvirus type 1	1 h	99%	23 ‐ 25	^44a^
					0.0125	Equine herpesvirus type 1	10 min	99%	23 ‐ 25	^44a^
					0.00175	Canine coronavirus (Strain S378)	3 days	3.0	37	^9^
	Didecyldimethyl ammonium chloride	Generally safe	Hospital, industrial, household disinfectants	Odorless, colorless, and non‐caustic. Requires warmer temperatures and longer reaction time. Virucidal effects unaffected by organic load.	0.0025	Canine coronavirus (Strain S378)	3 days	>4.0	37	^9^
					0.01	Equine herpesvirus type 1	10 min	> 99.99%	23 ‐ 25	^46^
					0.02	Equine herpesvirus type 1	5 min	> 99.99%	23 ‐ 25	^46^
					>0.02	Equine herpesvirus type 1	10 min	> 99.99%	0	^46^
	Mono; bis (tri‐methyl ammonium methylene chloride)‐alkyl (C_9‐15_) toluene	Generally safe	Hospital, industrial, household disinfectants	Odorless, colorless, and non‐caustic. Requires warmer temperatures and longer reaction time. Virucidal effects unaffected by organic load.	0.02	Equine herpesvirus type 1	10 min	> 99.99%	23 ‐ 25	^46^
					>0.02	Equine herpesvirus type 1	10 min	> 99.99%	0	^46^
Anionic Surfactants	Sodium linear alkylbenzene sulfonate	Generally safe	Usually used as a foaming agent in kitchen detergents for routine cleaning	Low cost. Virucidal effects reduced by organic load. Sensitive to water hardness.	0.0125	Equine herpesvirus type 1	10 min	>99.99%	23 ‐ 25	^46^
					0.025	Equine herpesvirus type 1	5 min	>99.99%	23 ‐ 25	^46^
					>0.05	Equine herpesvirus type 1	1 min	>99.99%	23 ‐ 25	^46^
					0.05	Equine herpesvirus type 1	10min	>99.99%	0	^46^
Non‐ionic Surfactants	Nonylphenoxy‐polyethoxy ethanol (Nonoxynol‐9)	High cytotoxicity	Mostly used as emulsifiers	High cytotoxicity and spermicidal, dilutions by >500 times required	0.01	Herpes Simplex Viruses ‐ 1	1 min	<500^×^	37	^44^
					0.01	Herpes Simplex Viruses ‐ 2	1 min	<500^×^	37	^44^
	*p*‐ diisobutylphenoxy‐polyethoxy ethanol (Triton X‐100)	High cytotoxicity	Mostly used as emulsifiers	High cytotoxicity and spermicidal, dilutions by >500 times required	0.002	Herpes Simplex Viruses ‐ 1	1 min	<500^×^	37	^44^
					0.002	Herpes Simplex Viruses ‐ 2	1 min	<500^×^	37	^44^
	Sorbitan monolaurate (Span‐20)	Lower cytotoxicity as compared to amide or ester bearing non‐ionic surfactants	Mostly used as emulsifiers	Lower cytotoxicity, formulation can be used without dilution	1	Herpes Simplex Viruses ‐ 1	1 min	8.8*10^6 ×^	37	^44^
					1	Herpes Simplex Viruses ‐ 2	1 min	6.1*10^5 ×^	37	^44^
	Polysorbate‐20 (Tween‐20)	Lower cytotoxicity as compared to amide or ester bearing non‐ionic surfactants	Mostly used as emulsifiers	Lower cytotoxicity, formulation can be used without dilution	1	Herpes Simplex Viruses ‐ 1	1 min	4.3*10^6 ×^	37	^44^
					1	Herpes Simplex Viruses ‐ 2	1 min	5.4*10^5 ×^	37	^44^
Halogenated compounds	Povidone‐iodine	Generally safe, long‐term exposure can affect thyroid function and	Hospitals, disinfecting handwashes, oral washes	Long‐lasting slow release of iodine, fast acting, more effective than many other disinfectants.	0.23	Influenza A subtype H1N1	15 s	5.67 ± 0.43	20.0	^141^
					0.023	Influenza A subtype H1N1	15 s	4.50 ± 0.54	20.0	^141^
					0.23	SARS‐CoV	15 s	4.60 ± 0.80	20.0	^141^
					0.23	MERS‐CoV	15 s	4.40 ± 0.79	20.0	^141^
					0.23	Rotavirus strain Wa	15 s	≥ 4.67 ± 0.42	20.0	^141^
					8	Vaccinia strain Elstree Belgium	30 s	≥ 4.21	‐	^101^
					8	Adenovirus Type 5	3 min	≥ 4.63	‐	^101^
					8	Polyomavirus SV40 Strain 777	30 s	≥ 4.29	‐	^101^
					8	Poliovirus Type 1	60 min	≥ 4.93	‐	^101^
	Chlorohexidine digluconate	Low skin irritability, safe, good skin persistence	Handwashes, mouthwashes and oral gels, disinfectants in hospitals^142^	Ineffective against non‐enveloped viruses. Less potent and slower‐acting than povidone‐iodine	0.02	Murine hepatitis virus	10 min	0.7 – 0.8	23	^143^
					0.12	Herpes‐simplex virus Type 1	30 s	97 %	A.T.	^108^
					0.12	Cytomegalovirus strain AD169	30 s	> 99.7 %	A.T.	^108^
					0.12	Influenza A	1 min	> 98 %	A.T.	^108^
					0.12	Parainfluenza Type 3	15 min	99 %	A.T.	^108^
					0.12	Hepatitis B virus	15 min	99 %	A.T.	^108^
	Chloroxylenol	Generally safe	Household disinfectants, cleaning of hospital surgical equipment	Can cause skin irritation, highly toxic to aquatic organisms	0.24	Herpes‐simplex virus Type 1	1 min	> 4.60	20	^22^
					0.24	HIV‐1	1 min	> 2.37	20	^22^
Aldehydes	Formaldehyde	Carcinogenic and irritant. Needs to be used in a well‐ventilated area.	Preservative, hostpital disinfecting agent	Pros: wide‐spectrum activity Cons: Pungent, hazardous to health	0.7	Murine hepatitis virus	10 min	> 3.45	23	^143^
					0.7	SARS‐CoV isolate FFM‐1	2 min	≥ 3.0	A.T.	^144^
					1.0	SARS‐CoV isolate FFM‐1	2 min	≥ 3.0	A.T.	^144^
					2.0	Vaccinia virus (ATTC VR‐1536)	2 h	4.9	4	^114^
					4.0	Vaccinia virus (ATTC VR‐1536)	3 h	8.2	25	^114^
					2.0	Human adenovirus Type 5	1 h	> 5.0	25	^114^
	Glutaraldehyde	irritant. Needs to be used in a well‐ventilated area.	Clinical settings only, not suitable for household disinfection	Pros: broad spectrum Cons: hazardous to health, deactivates by polymerization in alkaline media with time	0.5	SARS‐CoV isolate FFM‐1	2 min	> 5.0	A.T.	^144^
					0.1	Poliovirus Type 1	30 min	> 3.0	25	^124^
					0.1	Poliovirus Type 1	40 min	4.0	25	^124^
					0.05	Poliovirus Type 1	60 min	3.0	25	^124^
					0.02	Hepatitis A strain CF53	30 min	3.0	23	^123^
					0.1	Echovirus Type 25 JV‐4	5 min	> 2.0	25	^145^
Oxidising compounds	Sodium hypochlorite	Non‐flammable. Irritation to mucous membranes at high concentrations. Low incidence of serious toxicity. Toxic chlorine gas produced when mixed with acid	Cleaning and disinfection in hospitals, removing blood stain contamination	Pros: Broad spectrum, no toxic residues, fast‐acting Cons: Corrosive to metals at high concentrations, Decreased activity in the presence of organic matter	0.01	HIV‐1	30s	3.75	20	^82^
					0.016	Norwalk virus	30s	5.0	‐	^77^
					0.005	HIV‐1	2 min	∼3‐4	25	^83^
					0.005	Murine norovirus 3	1 min	∼2	‐	^73^
					0.001	PA01 bacteriophage F116	30s	>4.0	25	^24^
	Sodium dichloroisocyanurate	‐	‐	Pros: Broad‐spectrum, fast‐acting, long shelf life, easy to ship Cons: Strong smell, Some decrease in activity in the presence of organic matter,	0.005	HIV‐1	2 min	∼3‐4	25	^83^
	Hydrogen peroxide	inflammation due to insufficient rinsing	Disinfecting medical equipment	Pros: Broad‐spectrum, stable Cons: Slower acting	0.1	Feline calicivirus F9 (norovirus)	15 min	>3	20	^90^
	Peracetic acid	‐	‐	Pros: Fast acting, leaves no residue, still effective in organic matter Cons: corrodes metals, unstable	0.0085	Murine norovirus 3	1 min	∼3	‐	^73^

Abbreviations: AT, unspecified ambient temperature; RT, room temperature.

* Values given as log_10_ reduction factors unless otherwise specified.

The origins of the broad virucidal efficiencies of povidone‐iodine has still yet to be fully elucidated, and is likely to occur by multiple mechanisms, reducing the likelihood of chance viral mutations conferring resistance. There is evidence that iodine can block the receptors of the virus responsible for attachment to the host cell surface.^100^ Furthermore, iodine can inhibit the activity of viral enzymes (e.g. neuraminidase) essential for virus release from host cells, preventing its spread to other uninfected cells.^98^ For enveloped viruses, it has also been suggested that virus membrane can be destabilized by the reaction of iodine with the unsaturated C=C bonds of membrane fatty acids.^105^

#### 3.5.2 Chlorhexidine digluconate

Chlorhexidine is a broad‐spectrum cationic bisguanide biocide (Figure 5B) found in many antiseptic products. An active ingredient in handwashes, mouthwashes and oral gels (e.g., Corsodyl®), disinfectants, and preservatives, chlorhexidine has generally low irritability, good substantivity on skin, and rapid bactericidal activity. However, its activity is highly dependent on its formulation, being reduced by the presence of organic matter, including serum, as well as anionic surfactants and phospholipids^106^ as well as being pH‐dependent.^17^ Compared to bacteria, its virucidal activity is more variable and significantly less effective and slower‐acting than povidone‐iodine. Generally, chlorhexidine is ineffective against non‐enveloped viruses (polio and adenoviruses), but shows variable potency for inactivating enveloped viruses (e.g. herpes simplex virus, Influenza A, cytomegaloviruses and hepatitis B virus) (Table 3). Against human coronavirus HCoV‐299E however, chlorhexidine showed limited effectiveness, with a 1.0 mM solution showing only a 3 log_10_ reduction after 1 hour.^107^ Mechanistically, the viral inhibition of chlorhexidine has been proposed to arise from interactions with surface glycoproteins on enveloped viruses, which may reduce the activity of viral enzymes such as DNA polymerase enzymes for the hepatitis B virus.^108^ This chlorhexidine‐receptor binding is suggested to account for its effectiveness, albeit slow, against rotaviruses,^109^ which are non‐enveloped viruses containing surface glycoproteins that allow host cell infection.

#### 3.5.3 Chloroxylenol

Chloroxylenol, also known as para‐chloro‐meta‐xylenol (PCMX) (Figure 5C), is a halogenated phenolic‐type antiseptic used as the key active ingredient in Dettol®. Commonly‐used for household disinfectants, wound cleaning, and for disinfecting surgical equipment, it is most effective against bacteria, but its virucidal activity is variable. A 1998 study of chloroxylenol against a number of human viruses found it to be effective against the enveloped viruses herpes simplex 1 and HIV (Table 3), but was practically ineffective against the non‐enveloped polioviruses, adenoviruses and coxsackie virus, as well as the enveloped human coronavirus ATCC VR‐759.^22^ However, a later study using the murine hepatitis virus as a surrogate for the SARS coronavirus found it to be highly effective (≥4.50 log_10_ reduction) within a 30 s contact time.^111^ In spite of its widespread commercial use for a long time, surprisingly little is known about its mechanism of action against both bacteria and viruses. Chloroxylenol is generally safe to humans for external use, but has been reported to cause irritant contact dermatitis and contact depigmentation.^112^

### 3.6 Aldehydes

#### 3.6.1 Formaldehyde

**FIGURE 6:**
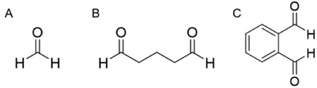
Chemical structures of (A) formaldehyde (B) glutaraldehyde and (C) ortho‐phthalaldehyde

Formaldehyde is the simplest aldehyde (Figure 6A) and a powerful high‐level disinfectant with potent viral inactivation capabilities. Often sold as an aqueous solution called formalin, it has been used to inactivate viruses for vaccine production^113^ and for scientific study^114^ As a high‐level disinfectant, it can effectively and quickly inactivate many different types of viruses (Table 3) both in suspension and on surfaces, by chemically alkylating the amino (NH_2_) and sulfhydryl (SH) groups of proteins,^115^ as well as the amino groups of nucleic acid bases (e.g. adenine) of DNA and RNA.^116^ As these functional groups are more reactive at alkaline pHs than acidic pH, formaldehyde is most effective as an alkaline solution. However, its high reactivity renders its usage health‐hazardous: other than being a mutagen and suspected carcinogen,^117^ it causes irritation of exposed body surfaces (e.g. skin and eyes).^118^, ^119^ Furthermore, its pungent odour can be detected at concentrations lower than 1 ppm. As a result, other than usage in a well‐ventilated area, strict regulations for human exposure govern its use as a disinfectant and sterilizing agent in hospitals and healthcare facilities, and thus it is not used as a household disinfectant. Formaldehyde has been shown to be a slower‐acting disinfectant than glutaraldehyde.^17^

#### 3.6.2 Glutaraldehyde

Like formaldehyde, glutaraldehyde (or sometimes known as glutardialdehyde) (Figure 6B), is a powerful broad‐spectrum disinfecting and sterilising agent which is highly effective against many viruses after short exposure times (Table 3). This dialdehyde is usually sold as an acidic solution, but its reactivity can be “switched on” by making the solution alkaline at pH > 7.5. In an alkaline solution, glutaraldehyde has a limited shelf‐life and stability, as its tendency to polymerise^120^ can reduce the number of reactive available free aldehyde groups essential for its virucidal activity. To determine the reactivity of glutaraldehyde solutions, chemical tests have been developed, though the manufacturer's instructions must be carefully adhered to for accurate results to be obtained.^121^ In a similar manner as formaldehyde, the aldehyde linkages of glutaraldehyde can react with the reactive groups on proteins, RNA and DNA. However, as a dialdehyde, its two reactive functional groups can form inter‐ and intra‐molecular crosslinks with these biomolecules that destroys their activity.^122^ For instance, glutaraldehyde inactivates the hepatitis A virus and enteroviruses by reacting with lysine residues on their surfaces.^123^ Reactions with capsid proteins are also proposed to account for its virucidal effectiveness against polioviruses.^124^, ^125^

Glutaraldehyde solution (2.0%) is usually used to decontaminate surgical equipment, endoscopes, and dialyzers in clinical settings, but it needs to be used in a well‐ventilated setting by trained personnel due to its strong and unpleasant odour. Although not suspected to be carcinogenic,^126^ it is known to cause dermatitis and irritation to mucous membranes in eyes, nose, and mouth.^127^ Due to these reasons, it is not used as a household disinfectant. Generally, metals, rubber, plastics, and lensed instruments are tolerant to glutaraldehyde, though it has been recommended not to be used to disinfect non‐critical surfaces due to its cost.^128^

#### 3.6.3 Ortho‐phthalaldehyde (OPA)

Ortho‐phthalaldehyde, or 1,2‐dicarboxybenzaldehyde (Figure 6C), is another high‐level disinfectant. Like both formaldehyde and glutaraldehyde, its virucidal properties stem from its reactions to crosslink reactive groups of proteins and nucleic acids. Although it is a less potent crosslinking agent than glutaraldehyde, this deficit is made up for by its more lipophilic aromatic nature which enhances its uptake by the lipid membrane, and has even shown faster bactericidal activity than glutaraldehyde.^129^ While OPA's bactericidal properties are well‐documented,^130^ its efficacies against viruses is less well established (Table 3). A study in 2006 using stainless steel careers showed that a 0.55% OPA solution could gave 4.84 log_10_ reduction in adenovirus 8 after 1 min exposure.^131^ 0.3% and 0.5% OPA solutions gave > 3 log_10_ reduction in the respective infectivities of surrogate hepatits B and C viruses on glass surfaces after a 1 min exposure.^132^ To assess the effectiveness of OPA against coronaviruses, a study using the surrogate mouse hepatitis virus on stainless stell surfaces showed 1.7 log_10_ reduction in infectivity after 1 min contact with 0.55% OPA.^133^ However, the same concentration of OPA was found to be ineffective against a suspension of human papillomavirus type 16 after a 45 min incubation.^134^ There us a current dearth of studies on the effectiveness of OPA against suspended viruses, and more studies are required to establish the general virucidal efficicacy of OPA against a wider variety of viruses.

OPA has numerous advantages over glutaraldehyde. First, it is chemically stable between pH 3 and 9 and does not require any further activation prior to usage. Second, it does not have a strong perceivable odour and does not irritate the skin, eyes, or nasal mucosa.^135^ As a result, exposure monitoring is not required for its usage, unlike glutaraldehyde. Furthermore, its excellent material compatibility^129^ allows its usage as a disinfectant in many clinical settings such as for endoscopes^135^, ^136^ and urological instruments. However, OPA can stain exposed skin grey, and hence has to be rinsed off with copious quantities of water, or used with personal protective equipment (e.g. gloves and eye protection). For this reason, it is not used as a common household disinfectant.

## 4 MYTHS ABOUT VIRUCIDAL AGENTS

The practice of maintaining adequate personal hygiene and environmental sanitation can be considered to be the single most consistent strategy that has been employed throughout the evolution of healthcare methods in combating the various epidemics the world has faced.^146^ Advisories issued by governmental agencies and the healthcare sectors alike have stressed its importance, ingraining in the minds of a wide audience the need for the procurement of viable sanitizing agents.

The global outbreaks of highly infectious viruses such as the SARS‐CoV‐2 have thus understandably seen rises in the mass purchasing of sanitizers and disinfectants, resulting in the issues of maintaining adequate supplies around several countries in the world. Without this option, a segment of the population has turned to the possibility of do‐it‐yourself formulations or alternative products that, along with the rise of social media, have largely been proliferated on the internet.

During the course of pursuing these options, it is of high imperative to consider the efficacies of these alternatives to ensure that ineffective options do not lull consumers into a false sense of security.

### 4.1 Essential Oils

Commonly used in a variety of skincare products to treat dermatological issues such as acne, essential oils were known to be both topically safe and able to combat a variety of skin‐associated pathogens. Yet, their explored germicidal activities are mostly bacteria‐related, and hence cannot be directly extrapolated into effective disinfection of all viruses.^147^

In terms of research into the anti‐viral or viral inhibiting efficacies of common essential oils, several oils have been proven effective against select viruses, such as the herpes simplex virus type‐1 or the bovine viral diarrhea virus.^148^ The operative mechanism of action, however, varies greatly between the different essential oils, and, depending on the stage of inactivation, render them effective against only certain specific strains.^149^

Through the study of star anise oil and some essential oil constituents, for example, it was identified that the oil itself, as well as the specific compounds trans‐anethole, farnesol and β‐caryophyllene were capable of direct inactivation of the herpes virus, potentially through the disruption of the virion envelope, prior to host cell infection.^149^ Other reports indicate that anti‐viral activities of essential oils do happen through similar mechanisms, including the virucidal effects of sesquiterpenes against various enveloped viruses,^150^ and the inhibitive effects of eugenol against the herpes simplex virus.^151^ Due to the lack of research investigating the specific virucidal actions of these compounds against the selected viruses, it is unsafe to assume that effective sanitization can occur broadly over all recommended types of essential oils. When considering several of the aforementioned cases as examples, most of which act upon the virion envelope, it is possible to conclude that they would be rendered ineffective in the disinfection of non‐enveloped virus species.

At the same time, it is also important to note that their purported antiviral activities may not be directly relevant to the purposes of sanitization or disinfection. Antiviral activities such as the inhibition of viral replication or gene expressions, while potentially effective in the treatment of viral infections, may not necessarily be what the user is expecting in terms of a topical or surface disinfectant.

### 4.2 Antibiotics

The development of global anti‐microbial resistance due to the over‐prescription of antibiotics is indicative of their high exposure to the public community. This could potentially allow for growth in public sentiment with regards to the belief that antibiotics are capable of treating a variety of diseases, including those caused by various viruses. An early survey of the public in the Netherlands showed that almost half of the population *incorrectly* believed in the ability of antibiotics in treating infections caused by viruses. In the same study, 90.9% believed in the need for antibiotic treatment when faced with pneumonia, and another study in a different area reported a 94.2% of the population sharing this belief.^152^, ^153^ This set of patient beliefs could have a negative impact on the decisions of healthcare professionals due to the pressure of catering to patient demands, even though it was shown to have no effect on the patient recovery and satisfaction.^154^

In fact, statistical evidence has shown that the inappropriate use of antibiotics prescribed by medical professionals has been observed to be high even up till recent years, where it was estimated that up to 30% of antibiotic prescriptions among outpatients may have been inappropriate.^155^ This could have potentially stemmed from a variety of reasons, including perceived patient expectation^154^ and misdiagnoses due to the similarities in the manifestations of clinical symptoms.^156^ As such, the correction of patient beliefs is still fundamental in the prevention of antibiotic overuse in such clinical settings.

### 4.3 Ultraviolet germicidal irradiation (UVGI)

There has been a long history of the employment of ultraviolet light in the elimination of microbial pathogens, whereupon its efficacy has been regarded highly enough for it to see use even in the disinfection in the laboratory and healthcare settings.^157^ It can thus be anticipated that there will be the resulting perpetuation of the notion that UVGI will be an effective agent in the combating of viruses. While this belief does hold merit, it is important to examine the restrictive conditions under which this does hold true.

First, as the efficacy of UVGI is dependent on the absorption by the target DNA, which has a maximum absorption wavelength of 260 nm, microorganisms have been found to be selectively vulnerable to the exposure to light at wavelengths specifically at or in close range of 253.7 nm.^158^ When tested against the strain of bacteriophage virus *Φ*X‐174, it was shown that the wavelength could be increased to up to 280nm without significant reduction of virucidal efficiency, but a significant *decrease* was observed upon the increase to 301 nm.^159^

Second, it has been shown that the germicidal activity of UVGI is compliant with the Bunsen–Roscoe reciprocity law, where it was established that the efficiency of inactivation is dependent on UV dose, which is the product of UV intensity (mW/cm^2^) and exposure time (seconds).^160^ The resulting implications in the translation into application mean that, upon a reduction of UV intensity, the virucidal action of UVGI can be compensated through the increase of exposure time.^161^ When tested for four different strains of airborne bacteriophages: ssRNA (MS2, ATCC 15597‐B1), ssDNA (*Φ*X‐174, ATCC 13706‐B1), dsRNA (phi 6 with envelope lipid, ATCC 21781‐B1), and dsDNA (T7, ATCC 11303‐B1), it was reported that the required UVGI dose required was approximately increased twofold when attempting to increase 90% inactivation to 99% inactivation.^162^ In the same study, it was also observed that certain strains of viruses were more vulnerable to UVGI than others. The UVGI doses required were, in increasing order, 339–423 µWs/cm^2^ for ssRNA, 444–494 µWs/cm^2^ for ssDNA, 662–863 µWs/cm^2^ for dsRNA, and 910–1196 µWs/cm^2^ for dsDNA.^162^ A similar ranking was reported when it came to observing the sensitivities of similar virus classes on a surface medium (as opposed to aerosol).^158^ The susceptibility of viruses to this method was thought to be dependent on several factors, such as physical size, molecular weight, DNA conformation, repair enzyme, and chromophore presence, clumping propensity, etc., which could all affect the UV dose required.^163^

As such, while there is a high potential for UVGI to serve as a virucidal technique, and even solar radiation to play a major role in environmental virucidal activity under appropriate conditions,^164^ it is important to consider the set of circumstances under which these are true.

### 4.4 Vitamin C

A popular dietary supplement, Vitamin C has had a history of being recommended in popular literature for the purposes of treating respiratory infections.^165^ While earlier studies have demonstrated its ability to prevent and alleviate the symptoms of virus‐induced respiratory infections, its mechanism of action, as well as its virucidal potency, were not examined.^165^

Prior to this, Vitamin C was the subject of various studies that investigated and concluded on its potential to inactivate the poliomyelitis virus under *in vivo* settings, but the route of its administration was either through injections^166^ or nasal instillation.^167^ It was however reported that, in this case, the dosage required was sufficiently low to be achieved via supplementation.^168^ Another *in* vivo study of viral inactivation involving the herpes virus also was achieved via injection.^169^ Apart from these, various other studies involving different virus strains, such as rabies^170^ and enteroviruses^171^, have reported *in vitro* viral inactivation.

The sum of gathered literature so far suggests, however, that the desired properties of ascorbic acid are achieved mainly through a combination of its ability to inactivate the virus, inhibit intracellular virus replication, as well as the other beneficial effects of the vitamins, which in turn render it capable of alleviating the severity of a range of virus‐related diseases.^168^ While indicative of the potential of the use of vitamin C to supplement medical therapy, this does not necessarily imply its potency as a virucidal disinfecting agent.

### 4.5 Garlic

The basis of some of these myths stems from their common association with antimicrobial properties. Garlic is commonly associated with fairly broad‐spectrum antimicrobial effects, exhibiting antibacterial, antifungal, antiviral and even antiprotozoal activities. Its constituents that render it effective against viruses have been discovered to include allicin, diallyl trisulfide, and ajoene.^172^, ^173^ It has displayed antiviral effects against a variety of viruses, including influenza A and B, rhinovirus, HIV, herpes simplex 2, cytomegalovirus, viral pneumonia, and rotavirus.^174^ In specifically considering its virucidal range, garlic has been shown to be effective against herpes simplex virus type 1 and the parainfluenza virus type 3.^173^ The virucidal effects of garlic and its active compounds do not cover the entire range of virus strains, and they have been shown to be ineffective against certain types, such as the coxsackievirus.^175^ High concentrations of garlic extract have also been shown to be toxic to cells, therefore caution is advised when garlic is attempted to be used.^175^ Thus, prior to turning to garlic as a viable alternative to conventional disinfectants, it is imperative to consider the strain of microbes to be eliminated, as well as whether garlic can be applied in a safe yet effective manner.

### 4.6 Saline Solutions

Apart from the aforementioned, there have also been various other myths circulating in the online community with regards to the prevention of the infections, especially with the case of the latest COVID‐19 pandemic, leading to the need for the clarification that has been conducted by the WHO and the official media of various countries. As mentioned in the previous segment, another commonly perpetuated belief is the inability of rinsing of the nose with saline solution, or gargling with salt water, to treat viral infections. Possibly due to its location of action, this is commonly associated with the treatment of upper respiratory tract infections and has a historical basis in the ancient practices from India.^176^

Upon investigation of its *in vivo* efficacy, it was shown that hypertonic saline nasal irrigation was capable of reducing the duration of illness by 22%, over the counter medicine use by 36% and illness in household members by 35%. Thirty percent more individuals also saw a reduction in viral shedding by ≥0.5 log_10_ per day, which could serve as a reason for the lowered transmission rates.^176^ The result shown, however, was not necessarily always positive, as some studies into the hypertonic saline nebulization of children with respiratory syncytial virus bronchiolitis showed no significant alleviation of their symptoms.^176^

The studies regarding saline solutions thus far, however, have mostly been investigations into its clinical effects, which, while somewhat suggestive of its potential in symptom reduction, are not sufficient to prove if it does possess virucidal behavior for disinfection purposes.

## 5 NEW RESEARCH DIRECTIONS TOWARDS VIRUCIDAL AGENTS AND MATERIALS

To expand the repertoire of virucidal compounds available, there has been considerable research effort to develop new active materials which exhibit broad spectrum virucidal activities, yet pose low toxicities to humans. Three main types of virucidal agents are receiving significant research attention: small discrete virucidal molecules (Section 5.1), metal nanomaterials (Section 5.2), and virucidal polymers (Section 5.3). There is a considerable push towards utilizing naturally‐occurring molecules to exploit their intrinsic virucidal properties as much as possible. In this section, we will give a broad overview of emerging directions towards virucidal agents and materials, which are not yet commercially available, and/or their virucidal properties are only demonstrated under controlled lab settings and not yet conclusively proven under real‐life usage conditions.

### 5.1 New virucidal molecules

**FIGURE 7:**
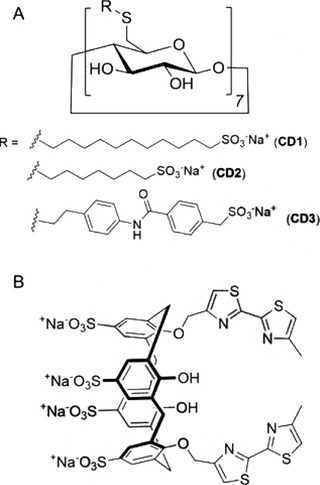
Structures of novel virucidal molecules: (A) β‐cyclodextrin alkyl sulfonates^177^ and (B) 1,3‐bis(bithiazolyl)‐tetra‐para‐sulfonato‐calix[4]arene.^107^

β‐cyclodextrins (βCDs) are naturally‐occurring macrocyclic molecules comprising of 7 covalently‐joined glucopyranose units, possessing a hydrophilic exterior and a hydrophobic interior cavity which can encapsulate non‐polar molecules in water. A family of sulfonated βCDs was recently studied for their virucidal activities (Figure 7A), which differed in the length of the flexible alkyl groups between the anionic sulfonate groups (CD1 and CD2), as well as rigidity of the spacer unit (CD3).^177^ CD1 displayed broad‐spectrum virucidal activity against many viruses from different families, including from herpes simplex virus type 2 (HSV‐2), respiratory syncytial virus types A and B, human metapneumovirus and human parainfluenza virus type A, while being ineffective against enterovirus D68 and influenza virus H3N2. The long alkyl linker of CD1 appeared to be the key to its high virucidal activity, giving complete HSV‐2 inactivation within 15 minutes, and whose virucidal activity was not affected by dilution. Compared to CD1, a shorter linker in CD2 and a more rigid one in CD3 made the cyclodextrin derivatives less effective. It was proposed that CD1's best virucidal activity against HSV‐2 stemmed from its ability to bind to most numbers of glycoprotein B found on the viral surface, blocking the proteins‘ fusion loop and induces conformational changes to these proteins.

In addition to cyclodextrin derivatives, macrocyclic calix[4]arene derivatives also showed promising virucidal activities. 1,3‐bis(bithiazolyl)‐tetra‐para‐sulfonato‐calix[4]arene (C[4]S‐BTZ) (Figure 7B) was shown to show better virucidal activities against human coronavirus HCoV‐229E than the frequently used antiseptic, chlorhexidine (CHX) (Section 3.5).^107^ Firstly, C[4]S‐BTZ were not cytotoxic towards L‐132 cells, whereas CHX and HXM showed notable toxicities with IC_50_ values of 4.3 × 10^−6^ and 3.8 × 10^−5^ mol/L, respectively. In addition, C[4]S‐BTZ demonstrated a faster virucidal rate as compared to CHX^107^. At a concentration of 10^−3^ mol/L, a 5 min‐incubation of HcoV‐229E with C[4]S‐BTZ reduced viral titers by 2.7 log_10_ units, as compared to just 1.4 log_10_ reduction for the same concentration and duration of CHX.

Interestingly, some compounds found in food were found to exhibit virucidal activities. Cinnamaldehyde, an organic compound that is responsible for cinnamon's flavor and odor, was effective against norovirus surrogates and hepatitis A virus^178^. Carvacrol, a natural monoterpene derivative of cymene, also known as a natural food additive was found to inactivate two norovirus surrogates completely at a concentration of only 0.5%.^179^ The grape seed extract was also shown to reduce the hepatitis A virus and feline calicivirus on infected lettuce and pepper after a minute of incubation^180^. These edible food extracts could be potentially used as virucidal agents in self‐sanitizing food packaging applications.

### 5.2 Metal nanomaterials

Silver and its salts have had a long history of use as antiseptics and disinfectants, and their broad‐spectrum biocidal properties are well established.^181^ Silver dihydrogen citrate, for instance, can reduce the infectivity of feline calicivirus, a surrogate for the human norovirus (which causes diarrhea and vomiting), by > 4 log_10_ units after 1 and 30 min in suspension and on glass surfaces, respectively.^182^ Silver nanoparticles (AgNPs) are a class of silver nanomaterials and are defined as dispersions of silver particles between 10 and 100 nm in size. Generally, AgNPs are effective biocides in small doses,^183^ although their potential toxicities to humans are still under intense debate.^184^, ^185^ Modern methods have enabled AgNPs of well‐defined shapes, particle sizes, and polydispersity to be synthesized,^186^ which are important parameters that dictate their eventual biocidal activities, biological fate and toxicity.^187^

The virucidal properties of AgNPs are still largely unexplored, but initial reports are encouraging. AgNPs can inhibit viruses by a number of mechanisms, including binding to and interacting with viral surface proteins,^185^ as well as denaturing enzymes by reacting with amino, carboxyl, imidazole, and sulfhydryl groups.^188^ In a study using 30–50 nm AgNPs surface‐coated with polyvinylpyrrolidone against various strains of HIV‐1, the AgNPs were found to bind onto surface glycoproteins (gp120) and chemically modifies it by denaturing its disulfide‐bonded domains.^189^ This prevents the virus from binding to receptor proteins on potential host cells which are necessary for viral entry and infection. Dilute AgNP solutions could elicit significant virucidal activity, as inhibition of 50 % of viral infectivity could be attained at AgNP concentrations of ≤ 0.91 mg/mL. Compared to commonly‐used biocidal silver nitrate and silver sulfadiazine, the AgNPs were found to be more effective against the HIV‐1 strains, suggesting that the release of Ag^0^ atomic clusters from the AgNPs is more potent virucide than Ag^+^ itself. Such destruction of viral surface glycoproteins has also been suggested to account for the virucidal effects of AgNPs against the Influenza virus.^190^ AgNPs have also been incorporated into polymer films comprising of poly (3‐hydroxybutyrate‐co‐3‐hydroxyvalerate), a biopolymeric material, which achieved the dual function of stabilizing the AgNPs and bringing about virucidal behavior against norovirus surrogates.^191^ AgNP‐containing products are appearing increasingly in the market, including clothing, wound‐dressings, ointments, and food packaging materials,^192^ whose biocidal activities are a result of slow sustained‐release of the silver nanomaterials. However, it should be noted that like all disinfecting agents aforementioned, the virucidal efficacies of AgNPs differ from virus to virus. Furthermore, the quantities, shapes, size, and types of silver nanomaterials released depend on their real‐world settings and applications,^193^ all of which affects their virucidal properties. Thus, the effectiveness of these AgNP‐containing products against viruses in real‐life settings, as well as their toxicity towards humans, need to be carefully evaluated and studied.

Other than AgNPs, gold nanoparticles (AuNPs) are also promising virucidal agents. AuNPs synthesized using garlic extract with an average size of 6 nm showed virucidal activity against the measles virus by also binding to surface viral receptors and preventing subsequent host cell binding and infection.^194^ However, due to the cost of the gold chemical precursors, AuNPs are unlikely to become cheap and commercially widely available disinfecting agents.

**FIGURE 8:**
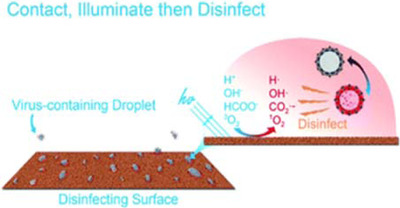
Illustration of virus disinfection using the self‐disinfecting surface powered by visible light. Figure reproduced from ref. 195 with permission from the Royal Society of Chemistry

The use of metal nanomaterials to form self‐disinfecting surfaces have gained traction in recent years, as viruses can persist on contaminated surfaces for prolonged periods. Self‐disinfecting surfaces are capable of inactivating viruses in contact with them *in situ*, reducing the chances of virus transmissions via human contact with contaminated surfaces. In one design, the self‐disinfecting surface was established with photoactive metal nanocrystals which required visible light stimulation for viral inactivation. These surfaces, fabricated from nanocrystals of CuInZn_4_S_6_ (CIZS) with band gaps within the visible light range, could absorb visible light and produce active oxidative species that inactivated the influenza A virus by oxidizing the amino acid residues presented on the viral envelope proteins (Figure 8). While highly virucidal, visible light must be present to guarantee the self‐sanitizing effect, thereby limiting the practicality of the system.

### 5.3 Polymers for inactivating viruses

Polymers capable of inactivating viruses are a new and exciting area of research. A large number of polymers capable of killing bacteria and preventing their proliferation are known today,^196^ but comparatively very few are known to be virucidal. Such polymers can: (1) act as carriers for controlled release of bioactive virucidal molecules, or (2) possess intrinsic viral inhibitory properties on their own due to the chemical functional groups present on the polymer structure. Compared to the former, whose virucidal efficiencies are limited by diffusion and initial loadings of the bioactive agents through the polymer matrix, the latter has the advantage of showing long‐term activity. Furthermore, these intrinsically viral‐inactivating polymers can be formulated or cast into various forms for customized applications, such as disinfecting coatings, binders in pharmaceutical products, water purification filters, and as additives in paper or common household materials. For these reasons, we discuss only intrinsically‐virucidal polymers here.

**FIGURE 9:**
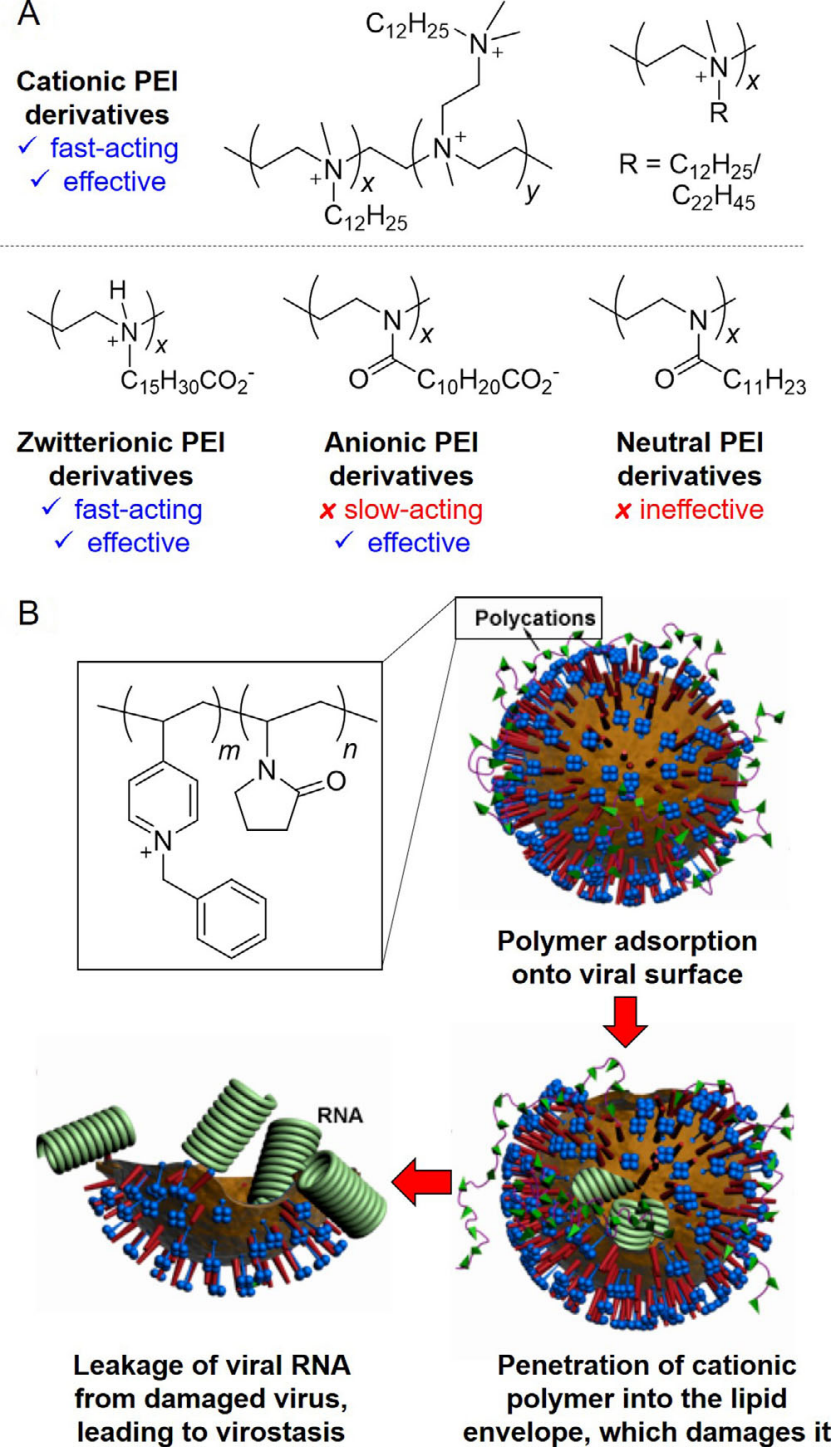
Inactivation of enveloped viruses by (A) hydrophobic charged PEI derivatives;^197^ and (B) pyridinium‐type polymers. Figure adapted from ref. 198. Counterions on the charged polymers are not shown

The vast majority of viral‐deactivating polymers are charged. In 2006, Klibanov reported that hydrophobic polyethylenimine (PEI) derivatives can inactivate enveloped viruses such as the Influenza A virus.^197^ Cationic and zwitterionic (possessing equal numbers of positive and negative charges) PEI derivatives (Figure 9A) showed close to complete the virucidal activity in 5 min. On the other hand, much slower virus inactivation was reported for anionic PEI, reaching 89 % virucidal activity only after 2 hr, while the neutral derivative showed no virus inactivation. Notably, an alcoholic solution of these polymers could be painted onto glass slides to form virucidal coatings. The inactivation was proposed to arise from the interaction of the erect charged polymer “tentacles” with ionic sites within the hydrophobic lipid membranes of the Influenza A virus. A similar mechanism of action likely accounted for the virucidal activity of cationic pyridinium‐type polyvinylpyrrolidones (Figure 9B), which damages the lipid envelop of Influenza A and leads to virostasis.^198^ Pyridinium polymers with a high degree of chemical crosslinking which are insoluble in water are also effective in removing viruses from water.^199^

**FIGURE 10:**
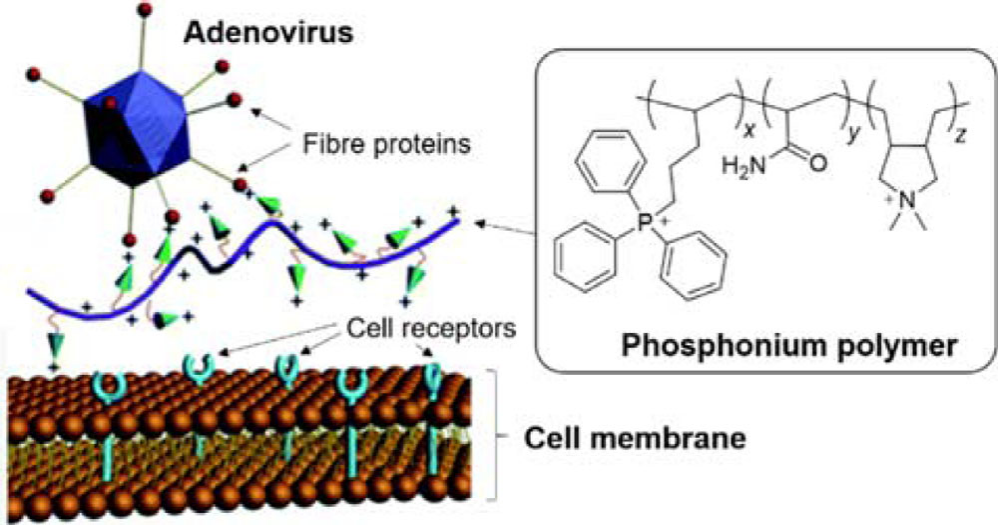
Inactivation of non‐enveloped adenoviruses using quarternary phosphonium polymers. Figure adapted from Ref.^200^ with permission from The Royal Society of Chemistry

The lack of a lipid membrane in non‐enveloped viruses (e.g. adenoviruses) necessitates a different strategy for inactivation. The cationic quaternary phosphonium polymer in Figure 10 achieves this by interacting with the binding fiber proteins on the virus, preventing them from binding to cellular receptors necessary for entry into cells. At aqueous concentrations as low as 100 ppm, these non‐cytotoxic polymers have a virucidal efficiency of 86.5%.^200^

**FIGURE 11:**
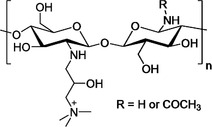
Cationic substituted chitosan polymers capable of inactivating human coronaviruses.^201^

In 2013, cationic chitosan derivatives (Figure 11) were shown to be highly potent for inhibiting the replication of human coronavirus HCoV‐NL63, a common cold virus.^201^ The combination of chitosan and the specific cationic quarternary ammonium substituent appears to be essential for its activity, as unfunctionalized chitosan, ι‐ and κ‐carrageenans and heparin were ineffective *in vitro* despite their ionic nature. The chitosan derivatives bearing different degrees of substitution were also highly selective in their virucidal activity, being effective against only murine hepatitis virus and a number of human coronaviruses (HCoV‐NL63, HCoV‐229E, HCoV‐OC43, and HCoV‐HKU1).^202^ Their virus inhibition occurred from the formation of a complex with the S protein of the coronaviruses, which prevents the viruses from binding to its cellular receptor for infection.

### 5.4 Disinfectants for inactivating airborne viruses

Airborne transmission of viruses is another major route of human‐to‐human transmissions and can occur in the form of aerosols (a cut‐off droplet size of < 5 µm is typically chosen).^203^ These aerosol microdroplets settle slowly from the air, and as long as the virus particles remain viable, are respirable and can result in direct viral transmission to the alveolar region. The airborne stability of viruses varies greatly, and depends on relative humidity and temperature. Lower humidity is generally favorable for viruses with more lipids, whilst viruses with low or no lipid content show stability at higher humidity.^204^ The effects of humidity on virus stability is also dependent on temperature‐ for instance, lower temperatures can enhance the stability of non‐enveloped rhinoviruses at high relative humidity.^205^ Airborne transmission is one of the major routes of transmission of the enveloped Influenza A virus,^206^ and has been implicated in SARS‐CoV‐1 superspreading events during the 2002–2004 outbreak.^207^ A recent study has also revealed that the Covid‐19‐causing SARS‐CoV‐2 virus possesses similar aerosol stabilities as the SARS‐CoV‐1, remaining viable for the 3‐hour duration of study.^16^ Furthermore, given the high momentum of the multiphase turbulent gas cloud emitted from sneezing and coughing, virus‐laden droplets may possibly be spread for distances larger than the 1–2 m recommended separation for SARS‐CoV‐2.^208^ In fact, SARS‐CoV‐2 virus particles were found in the ventilation systems of the hospital rooms housing Covid‐19 patients in China.^209^ Clearly, methods and disinfectants capable of disinfecting the air are thus valuable in limiting viral transmissions, especially within indoor environments.

Methods for disinfecting indoor air is currently an active field of research, though much more remains to be understood, and no perfect solutions yet exist. While UVGI can be effective and efficient in inactivating viruses in aerosols,^210^ it can also lead to significant skin and eye discomfort.^211^ Photocatalyst (silver ion‐doped TiO_2_)‐coated air filters^212^ and ionisers^213^ have also been recently studied and demonstrated to be effective in removing viable viruses from the air, though they are not expected to be stand‐alone solutions. Thus far, very few chemical disinfecting agents have been studied for inactivating airborne viruses.

In 2016, the use of extremely low concentrations of chlorine dioxide (ClO_2_) was reported to inactivate airborne viruses.^214^ Using the model bacteriophage viruses (MS2 and ΦX174), 0.01 and 0.02 ppm of ClO_2_ could reduce the number of viable bacteriophages by > 2 log_10_ units after 3 hours in an exposure chamber. Concentrations of ClO_2_ lower than 0.1 ppm has been shown to show no human toxicity. ClO_2_ is a stable free radical and is a powerful oxidizing agent, and can inactivate viruses by oxidizing a critical tryptophan residue in their binding site.^215^ Low concentrations of ClO_2_ has been found to decrease the infection of mice exposed to aerosols of influenza A virus,^216^ and instances of respiratory diseases in a Japanese army base,^217^ suggesting the possibility that the gas can control and limit airborne viral transmissions. However, more research to understand the long‐term effects of ClO_2_ exposure needs to be carried out.

## 6 CONCLUSION

The various classes of disinfectants/sanitizing agents inactivate viruses via different mechanisms and their potency against viruses is highly dependent upon the type of virus and application conditions such as concentration, exposure duration, temperature, pH, and organic load. Therefore, knowledge of the target virus and careful control of the application conditions are crucial to achieve effective disinfection. In general, it is observed that disinfectants/sanitizing agents like povidone‐iodine, aldehydes, and oxidizing agents that inactivate viruses by chemically modifying their surface groups are fast‐acting and highly potent towards most viruses but their application is also often limited by their higher toxicity and damaging effects to surfaces^101^, ^103^, ^118^, ^123^. On the other hand, disinfectants like alcohols and surfactants that mostly rely on dissolving the lipid envelopes tend to only show potency towards a narrower range of viruses and may require longer exposure durations, but are often more biocompatible^32^, ^42^. An ideal disinfectant/sanitizer would be one that is effective towards a broad range of viruses with fast‐action and high potency but still suitable for long‐term use, exhibiting good biocompatibility and mild effects towards surfaces. A potential direction may be to develop potent disinfectant agents from natural compounds^218^ as they may have less toxicity allowing the product to be child‐safe^219^ and also safe for long‐term usage. New generation sanitizers with viral inactivation mechanisms that can enhance and balance broad disinfection efficacy with biocompatibility are thus likely to have good potential in becoming the preferred choice of consumers.

## CONFLICT OF INTEREST

The authors declare no conflict of interest.
